# USEFULNESS OF PATIENT-GENERATED HEALTH DATA BY DEPRESSION AND FRAILTY IN OLDER ADULTS WITH LUNG CANCER SURGERY

**DOI:** 10.1093/geroni/igae098.2910

**Published:** 2024-12-31

**Authors:** Mona Choi, Yesol Kim, Hyeonmi Cho, Yeonju Kim

**Affiliations:** Yonsei University, Seoul, Republic of Korea; Yonsei University, Seoul, Republic of Korea; Yonsei University, Seoul, Republic of Korea; Yonsei University, Seoul, Republic of Korea

## Abstract

This study aimed to identify the perceived usefulness of patient-generated health data (PGHD) according to depression and frailty in older adults with lung cancer surgery. This cross-sectional study recruited patients aged 65 and over who were diagnosed with lung cancer and underwent lung resection at a tertiary hospital in the Republic of Korea. Depression was measured using the Center for Epidemiological Studies Depression Scale-10; frailty was assessed using the Korean FRAIL version and categorized into robust, prefrail, and frail. The perceived usefulness of PGHD was measured through 37 items from seven domains. Data were analyzed through one-way ANOVA, correlation, and linear regression analyses using the SPSS program. Among 132 participants, the average age was 71.79±5.20 years, with 53% men. The average depression score was 4.18±4.15 out of 30, and more than half were classified as robust (60.6%), 34.8% as prefrail, and 4.5% as frail. The higher the level of depression, the greater the perceived usefulness of Medication-related Factors (MF) among PGHD was identified (p=.028). Participants who were prefrail perceived the usefulness of Physical and Mental Well-being Factors (PMWF) (p=.015) and MF (p=.001) among PGHD to be higher compared to those who were robust. A higher perceived usefulness of PGHD was significantly associated with being prefrail (p=.032), older age (p=.017), and higher life satisfaction (p=.039). Our findings indicated that understanding the levels of depression and frailty in older adults after lung cancer surgery may help healthcare providers identify the perceived usefulness of PGHD and facilitate its application in research.

